# Perceptions and attitudes on biospecimen sample collection for dementia research in Kenya

**DOI:** 10.1002/alz70856_099672

**Published:** 2025-12-25

**Authors:** Lucy Wambui Kamau, Chinedu T Udeh‐Momoh, Karen Blackmon, Caroline Kalondu Kiio, Jasmit Shah, Vaibhav Narayan, Irene B Meier, Olivera Nesic‐Taylor, Zul Merali, Edna N Bosire

**Affiliations:** ^1^ Brain and Mind Institute, Aga Khan University, Nairobi, Kenya; ^2^ Wake Forest University School of Medicine, Winston‐Salem, NC, USA; ^3^ Geisel School of Medicine at Dartmouth, Hanover, NH, USA; ^4^ Aga Khan University, Nairobi, Kenya; ^5^ Davos Alzheimer's Collaborative, Wayne, PA, USA; ^6^ Davos Alzheimers Collaborative, Wayne, PA, USA; ^7^ Aga Khan University, The Brain and Mind Institute, Nairobi, Kenya; ^8^ Brain and Mind Institute, Aga Khan University, Nairobi, Nairobi, Kenya

## Abstract

**Background:**

Biospecimen sample collection is crucial in dementia diagnosis and evaluation. Limited research exists from African Countries including Kenya on people's perception on biospecimen sample collection for dementia‐ a disease that is largely unknown. Our aim was to understand the public perceptions towards collection of biospecimen samples for dementia research in Kenya.

**Method:**

We conducted 8 focused group discussions (FGDs; age and gender stratified) in Nairobi to understand people's perceptions about different biospecimens (urine, saliva, blood, tears, hair, cerebrospinal fluid (CSF) and the brain samples). An open‐ended FGD guide was used for the discussions, each lasting about 90 minutes.

**Result:**

Most participants were happy to provide blood and/or urine as biospecimens. There was heated discussion on whether saliva could be a sample for research, and many agreed that they could provide it. However, there were socio‐cultural concerns in relation to providing tears, hair, and brain for research – in part due to witchcraft practices. Participants were hesitant to provide CSF samples due to concerns such as (a) safety of the procedure; (b) the perceived pain; (c) qualification of research personnel involved in drawing the sample (d) sharing results from the procedure. Also, some participants demonstrated hesitancy on brain donation due to religious and cultural concerns.

**Conclusion:**

Biospecimen collection for dementia research is important and can enhance precise diagnosis of disease. There is a need to intensify education and advocacy on the importance of sample collection for dementia research. Communities must be engaged in dementia research to ensure that biospecimen collection and other clinical research procedures are appropriate and culturally acceptable

